# Construction of a resting EEG-based depression recognition model for college students and possible mechanisms of action of different types of exercise

**DOI:** 10.1186/s12888-023-05352-0

**Published:** 2023-11-16

**Authors:** Lili Li, Peng Wang, Shufan Li, Qun Zhao, Zhaosong Yin, Wei Guan, Sicheng Chen, Xing Wang, Jinlin Liao

**Affiliations:** 1 Department of Physical Education, Shanghai University of Engineering Science, Shanghai, China; 2 Shanghai University of Sport, Shanghai, China; 3 Department of Physical Education, Donghua University, Shanghai, China; 4 College of Physical Education and Health, Longyan University, Longyan, China

**Keywords:** College students, Depression, EEG, Exercise, Logistic regression, Mechanisms

## Abstract

**Objectives:**

To investigate the method of resting EEG assessment of depressive symptoms in college students and to clarify the relationship between physical activity level and depressive symptoms in college students.

**Methods:**

Using a cross-sectional study design, 140 current full-time college students were recruited to complete the Self-Rating Depression Scale and the International Physical Activity Questionnaire, and 10-min resting EEGs were obtained.

**Results:**

1) The power values of δ and α2 in the central (C3, C4) and parietal (P3, P4) regions of depressed college students were significantly higher than those of normal college students. And the degree of lateralization of δ, θ, α1, and α2 in the prefrontal regions (F3, F4) of depressed college students was significantly higher than that of normal college students (all *P* < 0. 008). 2) The recall rate of the depression recognition model for college students based on resting EEG was 66.67%, the precision was 65.05%, and the AUCs of the training group and validation group were 0.791 and 0.786, respectively, with better detection effects. 3) The two indicators, δ (C3 + C4) and α1 (F4-F3), are significantly correlated with IPAQ scores, and among college students who engage in ball games most commonly, those with a higher level of physical activity have lower δ (C3 + C4) and higher α1 (F4-F3), while among those who engage in resistance training most commonly, higher levels of physical activity are associated with lower δ (C3 + C4).

**Conclusion:**

The resting EEG of depressed college students has a certain specificity that can objectively assess the risk of developing depressive symptoms in college students. Physical activity is associated with abnormal EEG signals of depressive symptoms. Different types of physical activity may modulate the relationship between physical activity levels and EEG indicators.

## Introduction

Depression is the second most common human disease after coronary heart disease [1]. Studies show that the detection rate of depressive symptoms among college students in China is 24.71% [2], and depression is a common mental health problem among college students [3]. The main symptoms of depression in college students are reduced volitional activity, sleep disorders, low learning efficiency, interpersonal difficulties, and in severe cases, even self-harm or suicidal intention or behavior [4]. It is estimated that by 2030, depression will account for the first place in the world in terms of years lost due to disability [5].

Electroencephalography (EEG) recording is a non-invasive quantitative diagnostic tool for detecting rhythmic electrophysiological activity of neuron clusters in the cerebral cortex, and its signals can reflect mood-related physiological and pathological changes with the advantages of high temporal resolution and convenience [6, 7]. It has been found that there is a specificity in the EEG signals of people with depressive symptoms, such as low δ power during sleep [8, 9] and high β power during wakefulness [10–12], lateralization of left and right brain regions in θ and α waves [13–15] and asymmetry in power values of the high (β) and low (δ, θ) frequency bands [16]. Measuring depressive symptoms objectively and searching for EEG biomarkers in people with depressive symptoms has been one of the focuses of researchers [17–19].

Physical activity is associated with depressive symptoms, and insufficient physical activity is a risk factor for depressive symptoms [20, 21]. Studies reveal that exercise reduces the risk of depression in college students [22, 23]. Exercise negatively affects depressive symptoms. The EEG signal can provide a quantitative basis for exercise to alleviate depressive symptoms in college students. Exercise induces an immediate increase in cortisol after exercise, which affects brain oscillatory activity and alpha asymmetry of EEG. Exercise can effectively alleviate negative emotions in college students with depressive symptoms [24, 25]. However, the selection of subjects, exercise protocols, EEG frequency bands, and electrodes varied from study to study, as did the conclusions [26]. It has also been noted that existing studies are not yet sufficient to evaluate the effects of exercise interventions on EEG signals [27].

There is a negative correlation between physical activity and depressive symptoms, and EEG is a method to obtain pathological changes in the brain of people with depressive symptoms and to objectively assess depressive symptoms [19]. We found that there are still inconsistencies in the specific indicators of EEG in people with depressive symptoms, and it is particularly important to establish an EEG model to identify college students with depressive symptoms, to observe the relationship between physical activity levels, EEG indicators, and depressive symptoms, and to explore the moderating role of different types of physical activity in physical activity levels and EEG indicators. The present study adopts a cross-sectional research design and aims to clarify the specific indicators of EEG in college students with depressive symptoms, construct a model for detecting depression in college students based on resting EEG, clarify the relationship between physical activity level and depressive symptoms in college students, and reveal the possible mechanisms by which different types of physical activity affect depressive symptoms.

## Methods

### Study design and participants

This study used a cross-sectional research paradigm, and was approved by the Ethics Committee of Shanghai University of Sport (102772021RT007). All participants provided informed consent.

Inclusion criteria: (1) Participants must be willing to provide informed consent to participate in the study. (2) Participants must be undergraduate students aged between 18 and 22 years. (3) Participants must be right-handed. Exclusion Criteria: (1) Participants with a history of neurological disorders or head injuries that may affect EEG readings will be excluded. (2) Participants with physical diseases and dysplasia. (3) Participants who had used psychotropic drugs in the previous 3 months, or had consumed alcohol and caffeine in the previous 24 h as EEG measurement.

**Fig. 1:**
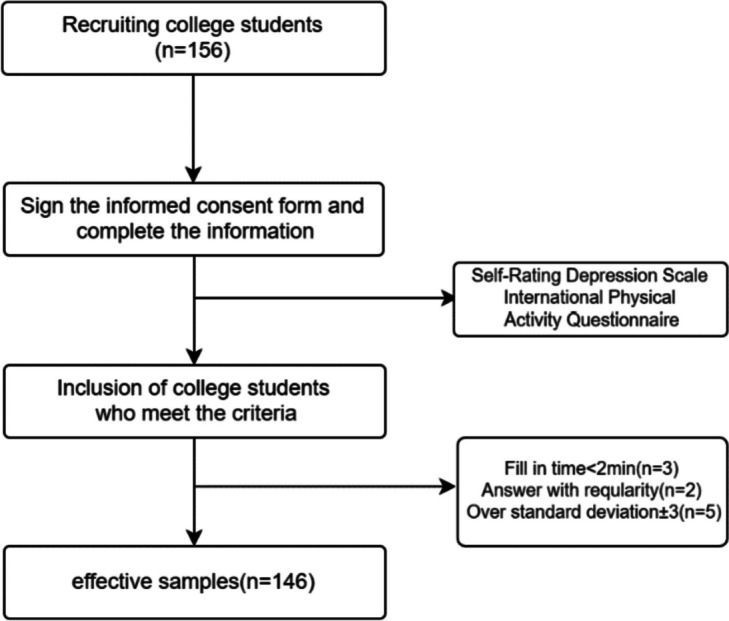
Recruiting flow chart

The measurement period was from November 2021 to April 2023. The recruitment process is shown in Fig. 1.

### Research methods

#### Questionnaire survey

The questionnaire was distributed to the subjects, in which it was clarified that the data obtained were used for scientific research only, and the principle of voluntary completion was adhered to. In the process of filling out the questionnaire, the subjects were actively guided to read the instructions carefully and prompted to complete the questionnaire carefully as required. After the questionnaire was completed, the investigators checked for logical errors or missing items to ensure that the information was filled in perfectly.

##### Self-Rating Depression Scale (SDS)

The Self-Rating Depression Scale (SDS) was developed by Professor Zung at Duke University School of Medicine, reflecting the depressed mood of the subjects over the last week. There are 20 items in the scale, and the answer options for each item are “no or little time”, “some of the time”, “good part of the time”, and “most of the time”, “Most of the time”, which are scored from 1 to 4 respectively. Ten of the entries are scored positively and the other 10 scored negatively. The scores of all entries are added together and multiplied by 1.25, which are rounded up to the standard score (score range 25–100). A standard score of < 53 is normal and ≥ 53 indicates depression. The internal consistency coefficient of this scale is 0.89.

##### International Physical Activity Questionnaire (IPAQ)

The questionnaire assesses the subjects’ exercise in the past week and classifies different physical activities into high, medium, and low intensity with metabolic equivalent (MET) values of 8.0, 4.0 and 3.3, respectively. The level of physical activity of a certain intensity = MET corresponding to that physical activity * frequency per week (day) * time per day (min). The sum of three intensity levels of physical activity is the total physical activity level. The criteria for classifying high physical activity are that the total of all types of high-intensity activities is greater than or equal to 3 days, and the total weekly physical activity level is greater than or equal to 1,500 MET, or the total of three types of physical activities is equal to 7 days, and the total weekly physical activity level is greater than or equal to 3,000 MET. Medium physical activity is classified as meeting the criteria of at least 20 min per day of all types of high-intensity activity combined greater than or equal to 3 days, or at least 30 min per day of all types of medium-intensity activity combined greater than or equal to 5 days, or three types of physical activity combined greater than or equal to 5 days, and the total weekly physical activity level greater than or equal to 600 MET. Low physical activity is classified as not reporting any activity or reporting some activity but not meeting the above criteria for medium and high grouping. The retest reliability coefficient of the International Physical Activity Questionnaire was 0.718 [28].

#### EEG signal acquisition

**Fig. 2:**
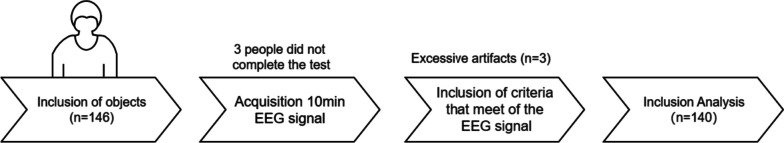
Test flow chart

The test period was 13:30–16:30, and the subjects performed resting EEG tests, and the test procedure is shown in Fig. 2.

EEG signals were recorded using the electroencephalograph (NCERP-190012) produced by Shanghai NCC Electric Co., Ltd., equipped with a preamplifier and 16 unipolar leads. Set the sampling frequency at 500 Hz, high-pass filtering at 0.3 Hz, low-pass filtering at 30 Hz, and trapping at 50 Hz. This instrument divides the EEG into delta band (1 to 4 Hz), theta band (4 to 8 Hz), alpha1 band (8 to 10.5 Hz), alpha2 band (10.5 to 13 Hz), beta1 band (13 to 20 Hz), and beta2 band (20 to 30 Hz) based on frequency.

**Fig. 3:**
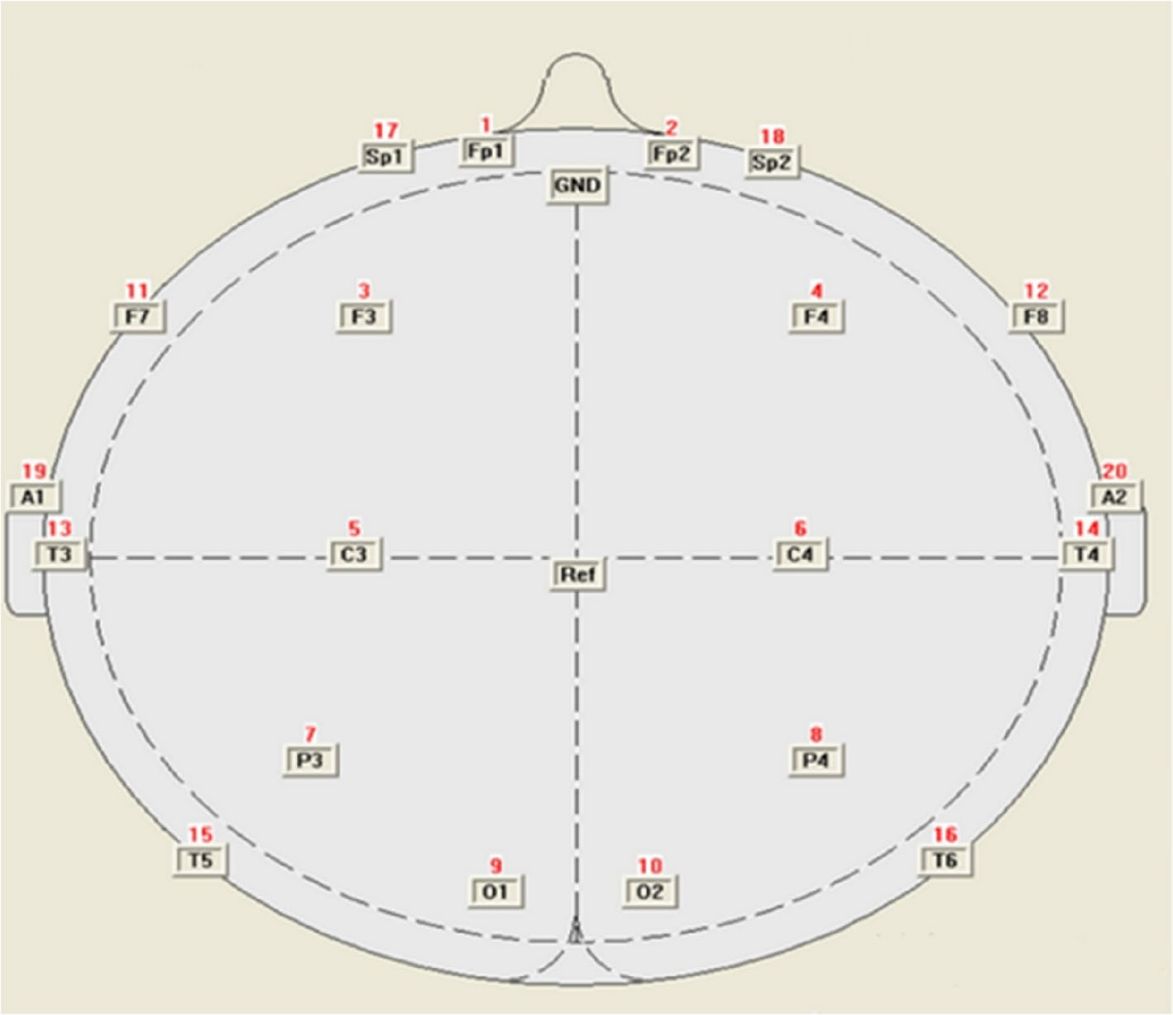
Diagram of brain electrode leads

The test environment was a quiet dark room. The subject was first seated in a chair and adjusted to a comfortable sitting position (Fig. 3). The tester set up Fp1, Fp2, F3, F4, C3, C4, P3, P4, F7, F8, O1, O2, T3, T4, T5, T6 leads according to the 10/20 system electrode placement method prescribed by the International EEG Society, with the ground electrode as Fpz and the reference electrodes as bilateral earlobes (A1 and A2) and tuned the impedance of each electrode to below 20 kΩ. At this point, the tester told the subjects to stay awake, relax their bodies as much as possible at rest, place their hands naturally at their sides, close eyes and do not clench their teeth or swallow saliva, sit still for 10 min to collect the EEG signal. The data segments of the first 2 min and the last 1 min and those with serious artifacts were removed.

#### Mathematical statistics

First, the EEG data were preprocessed. (1) The raw EEG signal data in edf format were imported into the EEGLAB tool in MATLAB and parsed into time series data in multiple channels. (2) We located the electrodes, removed the useless ones, and kept the Fp1, Fp2, F3, F4, C3, C4, P3, P4, F7, F8, O1, O2, T3, T4, T5, T6, A1, and A2 leads. Then, we selected the bilateral mastoid (A1 and A2) for re-referencing. (3) The ICA algorithm was utilized to eliminate artifacts such as ophthalmologic and electromyographic traces. (4) The spectrum was partitioned into distinct frequency bands, specifically setting the delta wave (1–4 Hz), theta wave (4–8 Hz), alpha1 wave (8–10.5 Hz), alpha2 wave (10.5–13 Hz), beta1 wave (13–20 Hz) and beta2 wave (20–30 Hz). (5) The frequency or power spectrum was computed using the Fourier transform (FFT) method. (6) The power values within each frequency band were calculated using the mean value calculation method for each electrode. (7) The signals in each lead have been categorized into appropriate brain regions, including orbital frontal (Fp1, Fp2), prefrontal (F3, F4), lateral frontal (F7, F8), central (C3, C4), parietal (P3, P4), occipital (O1, O2), temporal (T3, T4) and posterior temporal (T5, T6). The index of EEG power values for each brain region was determined by adding P left to P right. The index of EEG lateralization for each pair of homologous electrode sites in the left and right brain was calculated by (P right—P left)/(P left + P right). Higher values indicate greater right lateralization (P indicates absolute power value and left and right refer to the symmetrical electrode sites on the left and right sides of the brain).

Frequency histograms were utilized to observe data distribution. Measures that adhered to normal or approximate normal distribution were reported with mean ± standard deviation, and group comparisons were made through an independent samples t-test. Bonferroni was adopted to correct for multiple comparisons of frequency bands, with a corrected *P*-value of 0.05/6 = 0.0083. Median (interquartile range) was used to describe significantly skewed measures and group comparisons were conducted using the nonparametric Mann–Whitney U test. Count data were presented as n (%) and χ2 tests were used for group comparisons.

The dataset collected was randomly divided into training and validation cohorts at a ratio of 7:3, and the variables were compared. Non-normal data were presented as median (interquartile ranges). In the univariate analysis, chi-square test or Fisher’s exact test was used to analyze the categorical variables, while the Student’s t-test or rank-sum test was used to examine the continuous variables. In the training cohort, the least absolute shrinkage and selection operator (LASSO) logistic regression analysis was used for multivariate analysis to screen the independent risk factors and build a prediction nomogram for whether or not to be depressed. The performance of the nomogram was assessed using the receiver operating characteristic (ROC) curve and calibration curve, with the area under the ROC curve (AUC) ranging from 0.5 (no discriminant) to 1 (complete discriminant). A decision curve analysis (DCA) was also performed to determine the net benefit threshold of prediction. All statistical analyses were performed using the R software (version 4.2.2).

Pearson correlation analysis was used to explore the relationship between physical activity level and EEG indicators related to depressive symptoms, and to analyze the moderating effects of different types of physical exercise.

## Results

### Comparison of differences with depressive symptoms and normal college students

As depicted in Table 1, there were no notable differences in gender, age, BMI, and physical activity levels between depressed and normal college students. The power values of δ and α2 in the central (C3, C4) and parietal (P3, P4) regions of depressed college students were significantly higher than those of normal college students. And the degree of lateralization of δ, θ, α1, and α2 in the prefrontal regions (F3, F4) of depressed college students was significantly higher than that of normal college students (all *P* < 0. 008).

**Table 1:** Comparison of differences with depressive symptoms and normal college students

Variables	Depressive (*N* = 70)	Normal (*N* = 70)	Significant difference test
Sex (male%)	50%	50%	χ^2^ = 0, *P* > 0.999
Age	19.914 ± 1.213	20.043 ± 1.256	t = -0.616, *P* = 0.539
BMI (kg/m^2^)	22.292 ± 3.974	21.975 ± 3.062	t = 0.528, *P* = 0.598
IPAQ (MET-min/week)	1155.483 ± 897.210	1512.803 ± 1335.425	t = -1.858, *P* = 0.065
SDS	57.304 ± 3.876	40.679 ± 8.638	t = 14.691, *P* < 0.001
δ (FP1 + FP2)	3.094 ± 0.812	2.904 ± 0.900	t = 1.311, *P* = 0.192
θ (FP1 + FP2)	20.241 ± 4.068	19.980 ± 5.816	t = 0.308, *P* = 0.758
α1 (FP1 + FP2)	16.317 ± 6.216	15.314 ± 6.574	t = 0.927, *P* = 0.355
α2 (FP1 + FP2)	26.040 ± 7.802	23.074 ± 7.707	t = 2.263, *P* = 0.025
β1 (FP1 + FP2)	15.766 ± 3.451	15.306 ± 4.179	t = 0.710, *P* = 0.479
β2 (FP1 + FP2)	24.946 ± 6.540	25.326 ± 15.047	t = -0.194, *P* = 0.847
δ (F3 + F4)	2.610 ± 0.775	2.569 ± 0.733	t = 0.325, *P* = 0.746
θ (F3 + F4)	18.649 ± 6.278	19.021 ± 6.157	t = -0.355, *P* = 0.723
α1 (F3 + F4)	15.360 ± 7.368	15.401 ± 7.544	t = -0.033, *P* = 0.974
α2 (F3 + F4)	25.583 ± 9.668	23.630 ± 8.524	t = 1.268, *P* = 0.207
β1 (F3 + F4)	16.024 ± 4.647	15.666 ± 4.568	t = 0.460, *P* = 0.646
β2 (F3 + F4)	24.870 ± 8.239	25.407 ± 13.600	t = -0.283, *P* = 0.778
δ (C3 + C4)	2.906 ± 0.520	2.589 ± 0.586	t = 3.388, *P* = 0.001
θ (C3 + C4)	20.893 ± 4.743	19.524 ± 5.717	t = 1.542, *P* = 0.125
α1 (C3 + C4)	17.737 ± 6.787	16.079 ± 6.988	t = 1.425, *P* = 0.157
α2 (C3 + C4)	30.733 ± 10.094	25.979 ± 9.261	t = 2.904, *P* = 0.004
β1 (C3 + C4)	17.249 ± 3.774	16.299 ± 4.445	t = 1.363, *P* = 0.175
β2 (C3 + C4)	25.630 ± 6.166	26.150 ± 18.963	t = -0.218, *P* = 0.828
δ (P3 + P4)	3.214 ± 0.686	2.857 ± 0.823	t = 2.790, *P* = 0.006
θ (P3 + P4)	21.521 ± 5.516	20.499 ± 7.206	t = 0.943, *P* = 0.347
α1 (P3 + P4)	21.700 ± 10.388	19.249 ± 10.098	t = 1.416, *P* = 0.159
α2 (P3 + P4)	45.611 ± 20.820	35.961 ± 17.119	t = 2.995, *P* = 0.003
β1 (P3 + P4)	20.696 ± 5.252	19.054 ± 5.947	t = 1.731, *P* = 0.086
β2 (P3 + P4)	29.343 ± 6.842	28.540 ± 11.359	t = 0.507, *P* = 0.613
δ (O1 + O2)	3.421 ± 0.926	3.104 ± 1.069	t = 1.876, *P* = 0.063
θ (O1 + O2)	21.424 ± 5.651	20.687 ± 8.083	t = 0.625, *P* = 0.533
α1 (O1 + O2)	22.737 ± 10.860	21.717 ± 12.745	t = 0.510, *P* = 0.611
α2 (O1 + O2)	52.351 ± 23.400	44.160 ± 23.744	t = 2.056, *P* = 0.042
β1 (O1 + O2)	22.669 ± 6.095	21.603 ± 6.716	t = 0.983, *P* = 0.327
β2 (O1 + O2)	33.097 ± 9.799	34.121 ± 13.383	t = -0.517, *P* = 0.606
δ (F7 + F8)	2.471 ± 0.472	2.314 ± 0.658	t = 1.624, *P* = 0.107
θ (F7 + F8)	16.484 ± 3.571	16.037 ± 4.750	t = 0.630, *P* = 0.530
α1 (F7 + F8)	12.679 ± 5.192	12.191 ± 5.722	t = 0.528, *P* = 0.599
α2 (F7 + F8)	21.500 ± 6.697	19.019 ± 7.495	t = 2.066, *P* = 0.041
β1 (F7 + F8)	12.783 ± 2.999	12.424 ± 3.424	t = 0.659, *P* = 0.511
β2 (F7 + F8)	20.060 ± 5.527	20.104 ± 9.420	t = -0.034, *P* = 0.973
δ (T3 + T4)	2.051 ± 0.470	1.957 ± 0.641	t = 0.992, *P* = 0.323
θ (T3 + T4)	13.799 ± 3.284	13.616 ± 4.881	t = 0.260, *P* = 0.795
α1 (T3 + T4)	11.484 ± 5.085	11.276 ± 5.820	t = 0.226, *P* = 0.822
α2 (T3 + T4)	21.414 ± 7.363	20.176 ± 10.965	t = 0.785, *P* = 0.434
β1 (T3 + T4)	14.547 ± 4.095	13.941 ± 4.852	t = 0.798, *P* = 0.426
β2 (T3 + T4)	23.044 ± 8.393	23.136 ± 14.557	t = -0.046, *P* = 0.964
δ (T5 + T6)	3.009 ± 0.732	2.701 ± 1.030	t = 2.033, *P* = 0.044
θ (T5 + T6)	19.494 ± 5.268	18.600 ± 7.953	t = 0.784, *P* = 0.434
α1 (T5 + T6)	20.280 ± 9.890	19.083 ± 12.091	t = 0.641, *P* = 0.522
α2 (T5 + T6)	41.717 ± 17.857	34.723 ± 17.110	t = 2.366, *P* = 0.019
β1 (T5 + T6)	18.659 ± 4.647	17.437 ± 5.114	t = 1.479, *P* = 0.141
β2 (T5 + T6)	26.119 ± 6.830	26.691 ± 12.651	t = -0.333, *P* = 0.739
δ (FP2-FP1)	-0.007 ± 0.092	-0.007 ± 0.068	t = -0.004, *P* = 0.996
θ (FP2-FP1)	-0.015 ± 0.057	-0.003 ± 0.051	t = -1.365, *P* = 0.175
α1 (FP2-FP1)	-0.014 ± 0.072	-0.005 ± 0.045	t = -0.928, *P* = 0.355
α2 (FP2-FP1)	-0.004 ± 0.060	0.012 ± 0.040	t = -1.908, *P* = 0.059
β1 (FP2-FP1)	0.010 ± 0.053	0.024 ± 0.045	t = -1.682, *P* = 0.095
β2 (FP2-FP1)	0.024 ± 0.061	0.039 ± 0.070	t = -1.431, *P* = 0.155
δ (F4-F3)	-0.048 ± 0.083	0.011 ± 0.076	t = -4.395, *P* < 0.001
θ (F4-F3)	-0.034 ± 0.045	0.006 ± 0.062	t = -4.374, *P* < 0.001
α1 (F4-F3)	-0.040 ± 0.075	0.017 ± 0.062	t = -4.868, *P* < 0.001
α2 (F4-F3)	-0.031 ± 0.073	0.024 ± 0.063	t = -4.739, *P* < 0.001
β1 (F4-F3)	-0.004 ± 0.098	0.033 ± 0.068	t = -2.582, *P* = 0.011
β2 (F4-F3)	-0.014 ± 0.130	0.029 ± 0.087	t = -2.335, *P* = 0.021
δ (C4-C3)	-0.034 ± 0.076	-0.005 ± 0.077	t = -2.192, *P* = 0.030
θ (C4-C3)	-0.027 ± 0.074	-0.006 ± 0.069	t = -1.701, *P* = 0.091
α1 (C4-C3)	-0.024 ± 0.064	0.002 ± 0.078	t = -2.090, *P* = 0.039
α2 (C4-C3)	-0.008 ± 0.049	0.022 ± 0.080	t = -2.665, *P* = 0.009
β1 (C4-C3)	0.004 ± 0.061	0.026 ± 0.059	t = -2.181, *P* = 0.031
β2 (C4-C3)	0.006 ± 0.072	0.028 ± 0.080	t = -1.720, *P* = 0.088
δ (P4-P3)	-0.010 ± 0.096	0.022 ± 0.059	t = -2.372, *P* = 0.019
θ (P4-P3)	-0.009 ± 0.080	0.020 ± 0.060	t = -2.426, *P* = 0.017
α1 (P4-P3)	-0.001 ± 0.059	0.020 ± 0.063	t = -2.035, *P* = 0.044
α2 (P4-P3)	0.007 ± 0.067	0.030 ± 0.078	t = -1.814, *P* = 0.072
β1 (P4-P3)	0.016 ± 0.060	0.032 ± 0.054	t = -1.690, *P* = 0.093
β2 (P4-P3)	0.024 ± 0.074	0.033 ± 0.066	t = -0.767, *P* = 0.445
δ (O2-O1)	-0.018 ± 0.085	0.002 ± 0.076	t = -1.511, *P* = 0.133
θ (O2-O1)	-0.016 ± 0.075	0.001 ± 0.071	t = -1.384, *P* = 0.169
α1 (O2-O1)	-0.011 ± 0.075	-0.002 ± 0.081	t = -0.717, *P* = 0.475
α2 (O2-O1)	0 ± 0.080	0.001 ± 0.094	t = -0.028, *P* = 0.978
β1 (O2-O1)	0.016 ± 0.061	0.006 ± 0.099	t = 0.704, *P* = 0.483
β2 (O2-O1)	0.036 ± 0.091	0.018 ± 0.125	t = 0.985, *P* = 0.327
δ (F8-F7)	-0.007 ± 0.107	-0.021 ± 0.112	t = 0.793, *P* = 0.429
θ (F8-F7)	-0.017 ± 0.092	-0.019 ± 0.093	t = 0.078, *P* = 0.938
α1 (F8-F7)	-0.024 ± 0.077	-0.008 ± 0.074	t = -1.258, *P* = 0.211
α2 (F8-F7)	-0.030 ± 0.076	-0.006 ± 0.076	t = -1.925, *P* = 0.056
β1 (F8-F7)	-0.019 ± 0.074	0.005 ± 0.068	t = -1.956, *P* = 0.053
β2 (F8-F7)	-0.018 ± 0.095	0.006 ± 0.108	t = -1.346, *P* = 0.181
δ (T4-T3)	-0.029 ± 0.152	-0.002 ± 0.144	t = -1.079, *P* = 0.282
θ (T4-T3)	-0.037 ± 0.118	-0.005 ± 0.121	t = -1.615, *P* = 0.108
α1 (T4-T3)	-0.018 ± 0.111	-0.006 ± 0.137	t = -0.568, *P* = 0.571
α2 (T4-T3)	-0.009 ± 0.115	0.008 ± 0.165	t = -0.718, *P* = 0.474
β1 (T4-T3)	0.010 ± 0.149	0.021 ± 0.148	t = -0.416, *P* = 0.678
β2 (T4-T3)	0.012 ± 0.190	0.019 ± 0.152	t = -0.235, *P* = 0.814
δ (T6-T5)	0.026 ± 0.128	0.039 ± 0.109	t = -0.636, *P* = 0.526
θ (T6-T5)	0.009 ± 0.106	0.027 ± 0.102	t = -1.002, *P* = 0.318
α1 (T6-T5)	0.031 ± 0.132	0.049 ± 0.129	t = -0.779, *P* = 0.437
α2 (T6-T5)	0.061 ± 0.148	0.075 ± 0.166	t = -0.546, *P* = 0.586
β1 (T6-T5)	0.041 ± 0.099	0.056 ± 0.106	t = -0.861, *P* = 0.391
β2 (T6-T5)	0.056 ± 0.107	0.064 ± 0.128	t = -0.423, *P* = 0.673

### Construction of a resting EEG-based depression recognition model for college students

#### Construction of depression recognition model

The dataset collected was randomly divided into training and validation cohorts at a ratio of 7:3. Basic information is shown in Table 2, and there is homogeneity between the two groups.

**Table 2:** Patient demographics and baseline characteristics

Characteristic	Training Cohort, *N* = 98	Test Cohort, *N* = 42	*p*-value
Sex (male%)	51 (52%)	19 (45%)	0.461
Age	20.16 ± 1.15	19.79 ± 1.02	0.057
BMI (kg/m^2^)	22.04 ± 3.18	22.41 ± 4.17	0.563
IPAQ (MET-min/week)	1366 ± 1209	1260 ± 998	0.593
SDS	49 ± 11	49 ± 11	0.797

**Fig. 4:**
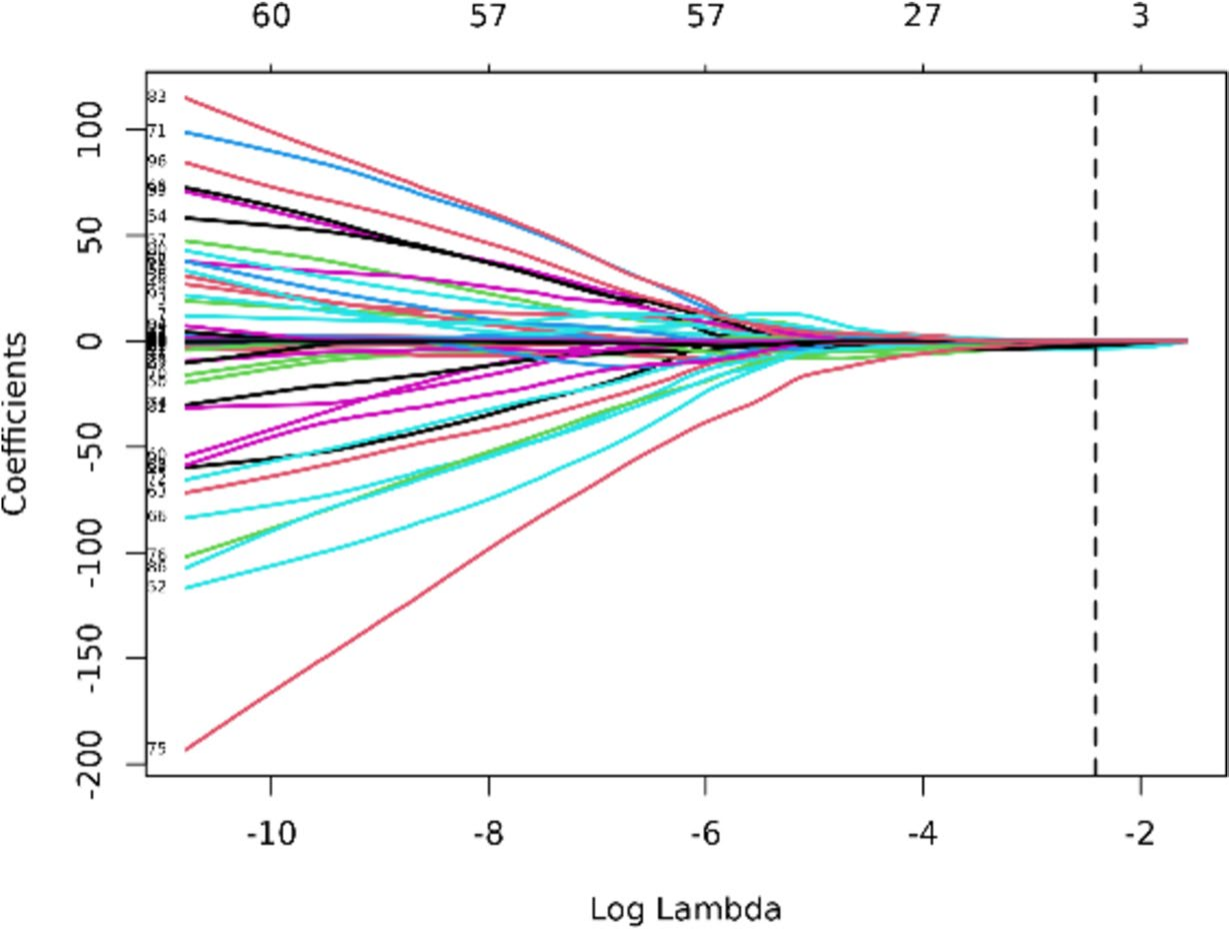
Lasso regression coefficient path plot

**Fig. 5:**
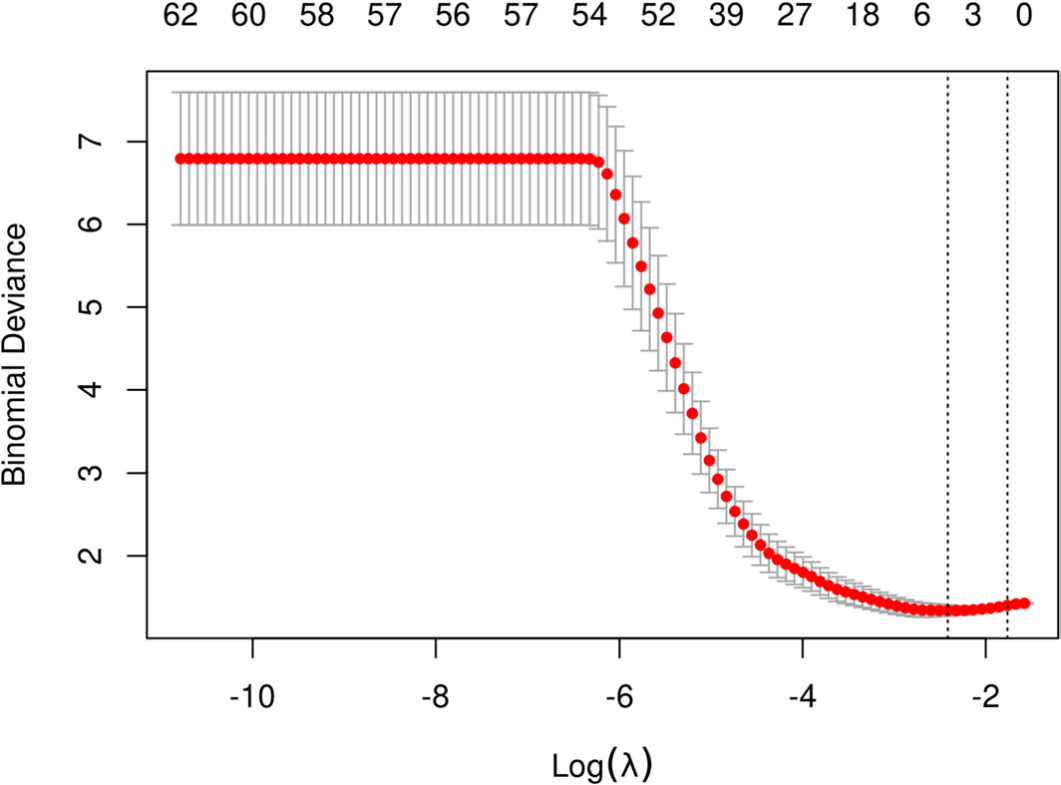
Lasso regression cross-validation plot

**Fig. 6:**
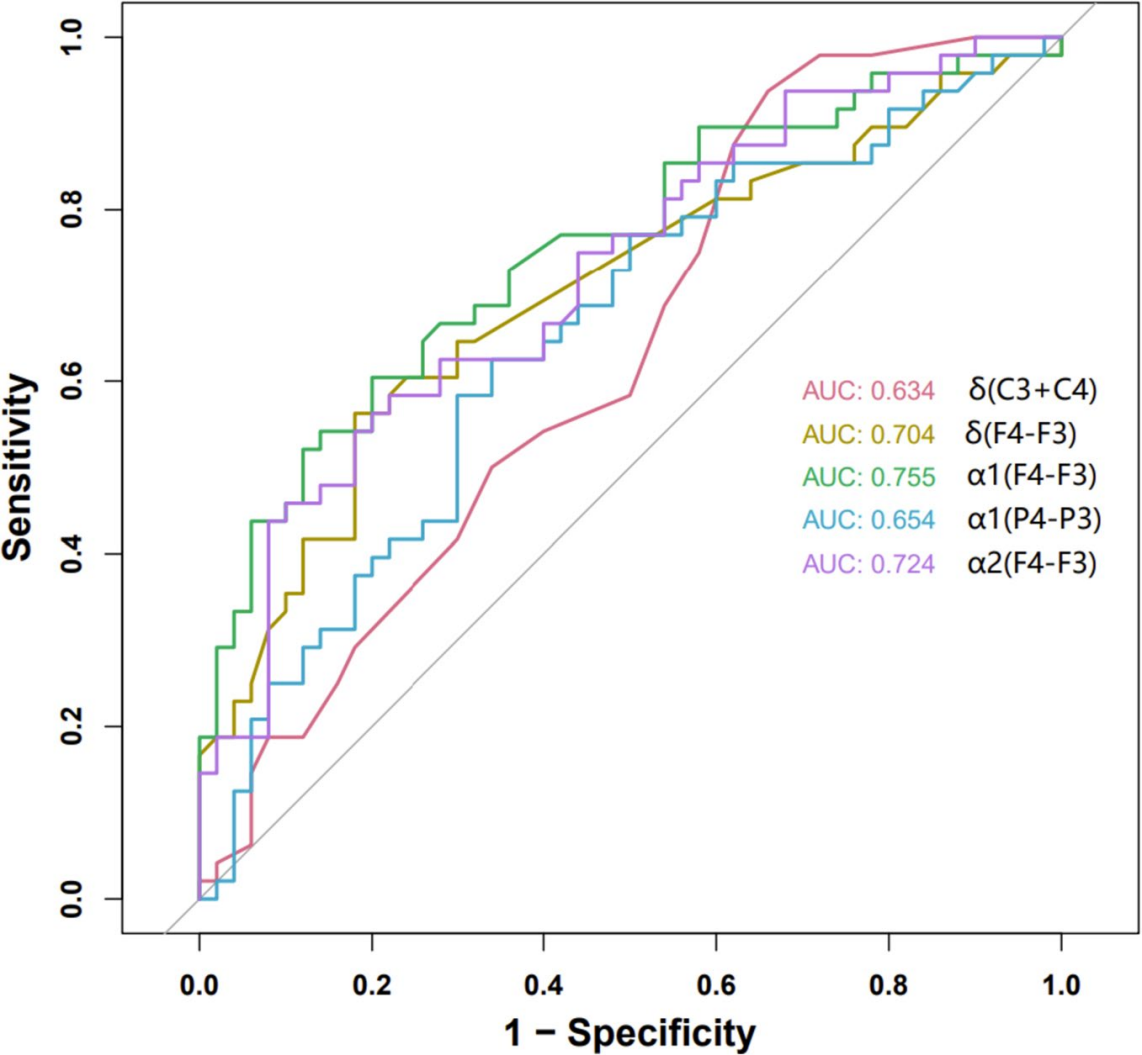
ROC curve analysis of 5 candidate diagnostic indicators

All EEG indicators as candidate predictors were included in the original model, which were then reduced to 5 potential predictors using LASSO regression analysis performed in the training cohort. The coefficient profile is plotted in Fig. 4 and cross-validated error plot of the LASSO regression model is also shown in Fig. 5. The most regularized and parsimonious model, with a cross-validated error within one standard error of the minimum, included 5 variables. ROC curves for each factor are shown in Fig. 6.

**Fig. 7:**
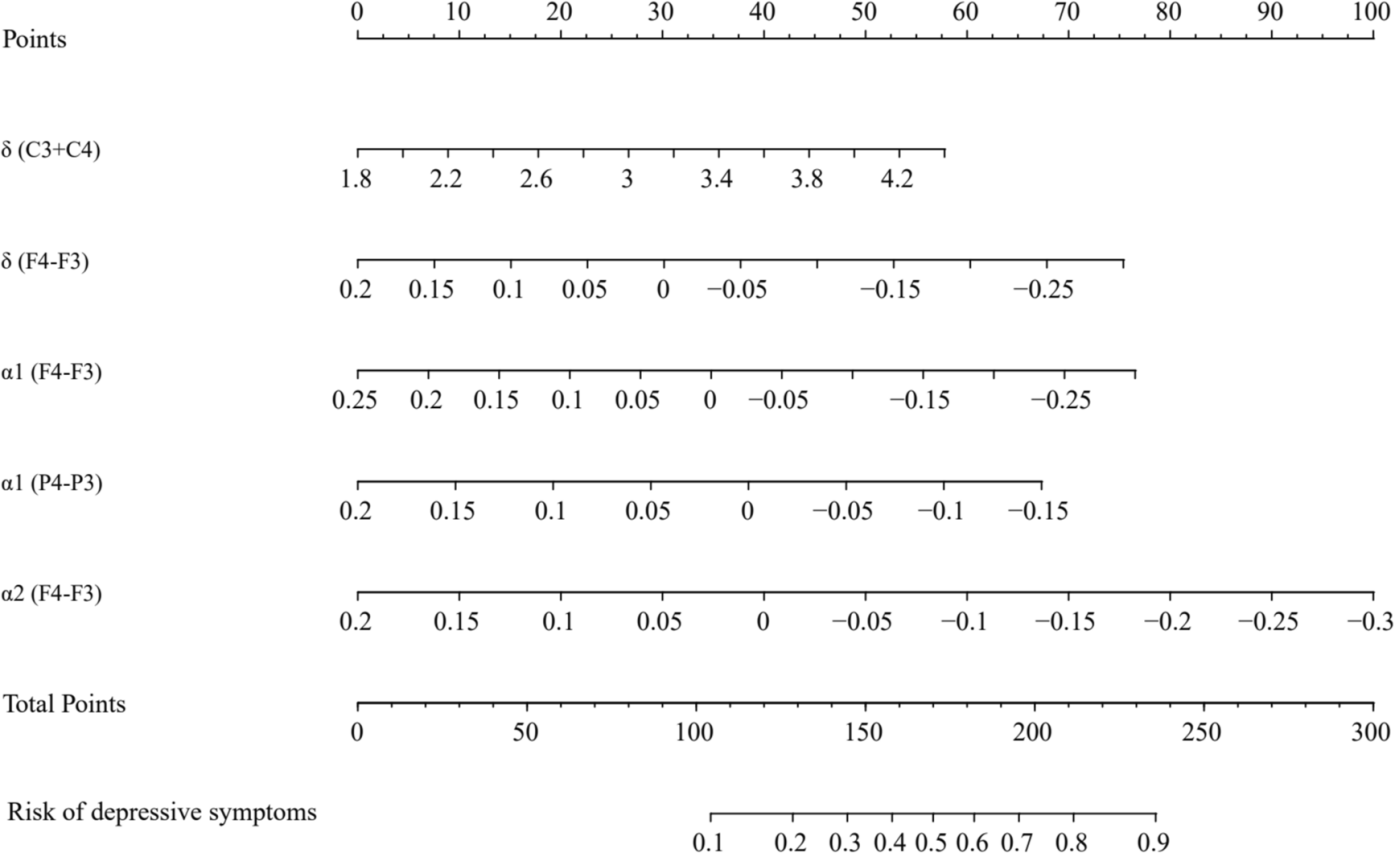
Nomogram prediction model

Further multivariate logistic analyses were carried out in different cohorts. Results are shown in Table 3. The final logistic model included 5 independent predictors and was developed as a simple-to-use nomogram, which is illustrated in the Fig. 7.

**Table 3:** Results of multivariate logistic regression for training cohort

EEG indicators	N	Event N	OR	95% CI	*p*-value
δ (C3 + C4)	98	48	2.10	0.92, 5.09	0.085
δ (F4-F3)	98	48	0.01	0.00, 2.60	0.108
α1 (F4-F3)	98	48	0.01	0.00, 305.15	0.339
α1 (P4-P3)	98	48	0.00	0.00, 2.29	0.091
α2 (F4-F3)	98	48	0.00	0.00, 30.56	0.202

*OR* Odds Ratio, *CI* Confidence Interval

#### Evaluation of the testing effectiveness of the model

**Fig. 8:**
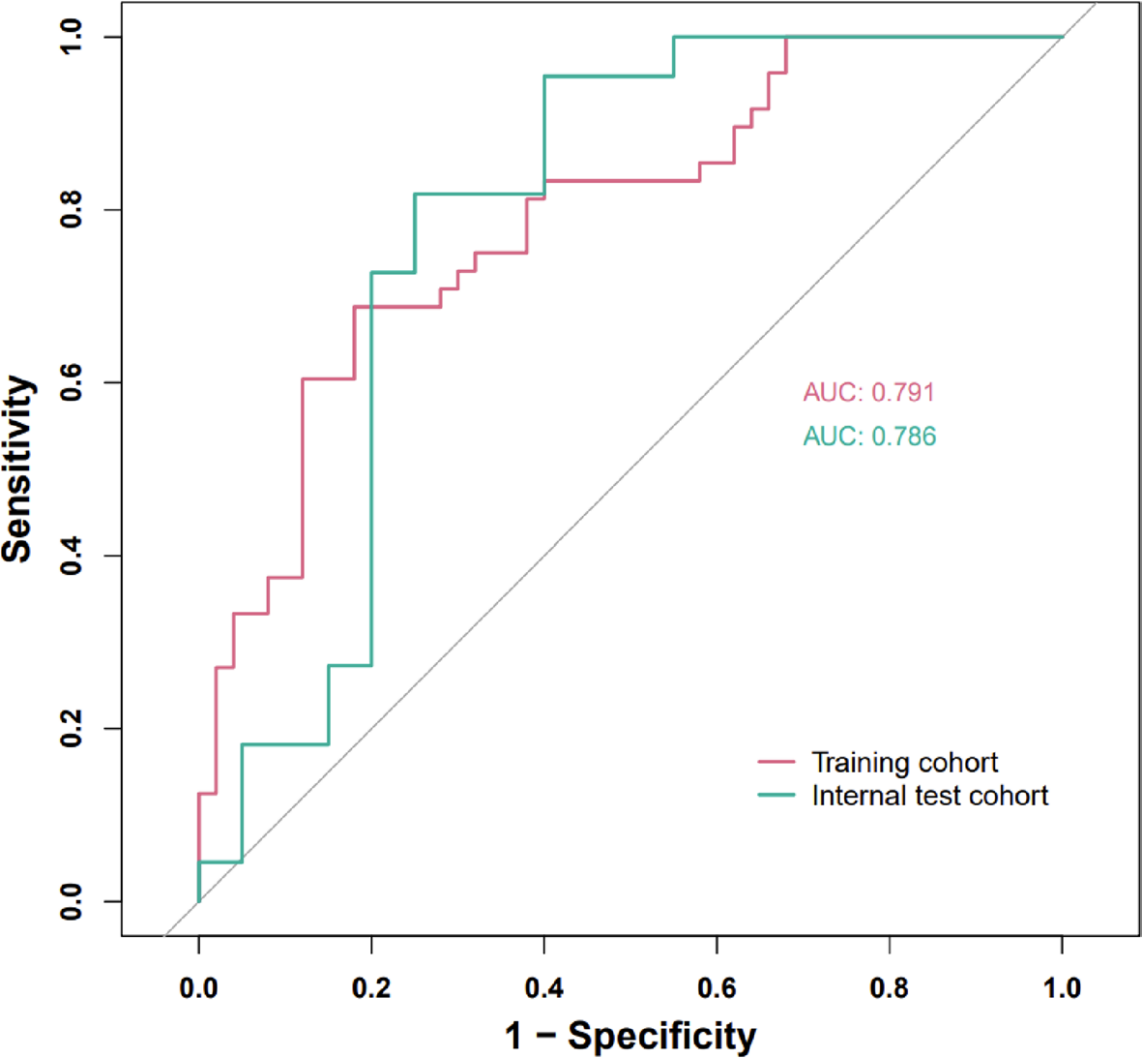
ROC curves of the nomogram prediction model

As shown in Table 4, The self-fitting confusion matrix of this recognition model revealed that 21 college students with depressive symptoms were correctly identified in 14 cases and incorrectly identified in 7 cases. Twenty-three normal college students were correctly identified in 15 cases and incorrectly identified in 8 cases, with a recognition recall of 66.67% and a precision of 69.05% (Table 4). The AUCs of the model in the different cohorts were shown in Fig. 8.

**Table 4:** Confusion matrix for recognition models

	Recognition of depression	Recognition of normal
Depressive Symptoms (*n* = 21)	14 (True Positive)	7 ( (False Negative)
Normal (*n* = 23)	8 (False Positive)	15 (True Negative)

The recall rate = true positive / (true positive + false negative), precision = true positive / (true positive + false positive)

**Fig. 9:**
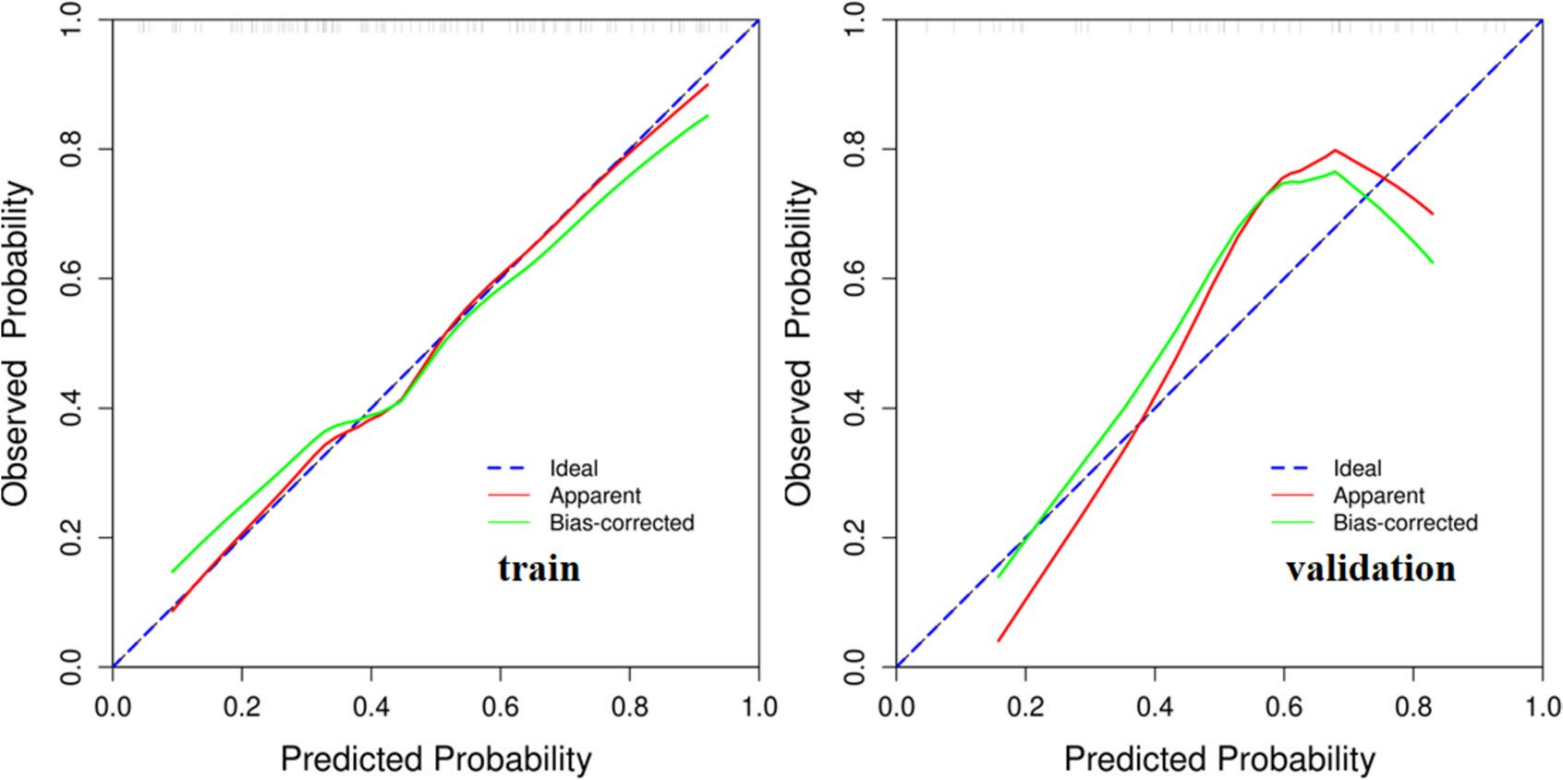
Calibration curve of the nomogram prediction mode

The internal validation and calibration of the nomogram were performed using 1,000 bootstrap analyses. The calibration plots of the nomogram in the different cohorts are plotted in Fig. 9, which demonstrate a good correlation between the observed and predicted whether depressive symptoms are detected. The results showed that the original nomogram was still valid for use in the validation sets, and the calibration curve of this model was relatively close to the ideal curve, which indicates that the predicted results were consistent with the actual findings.

**Fig. 10:**
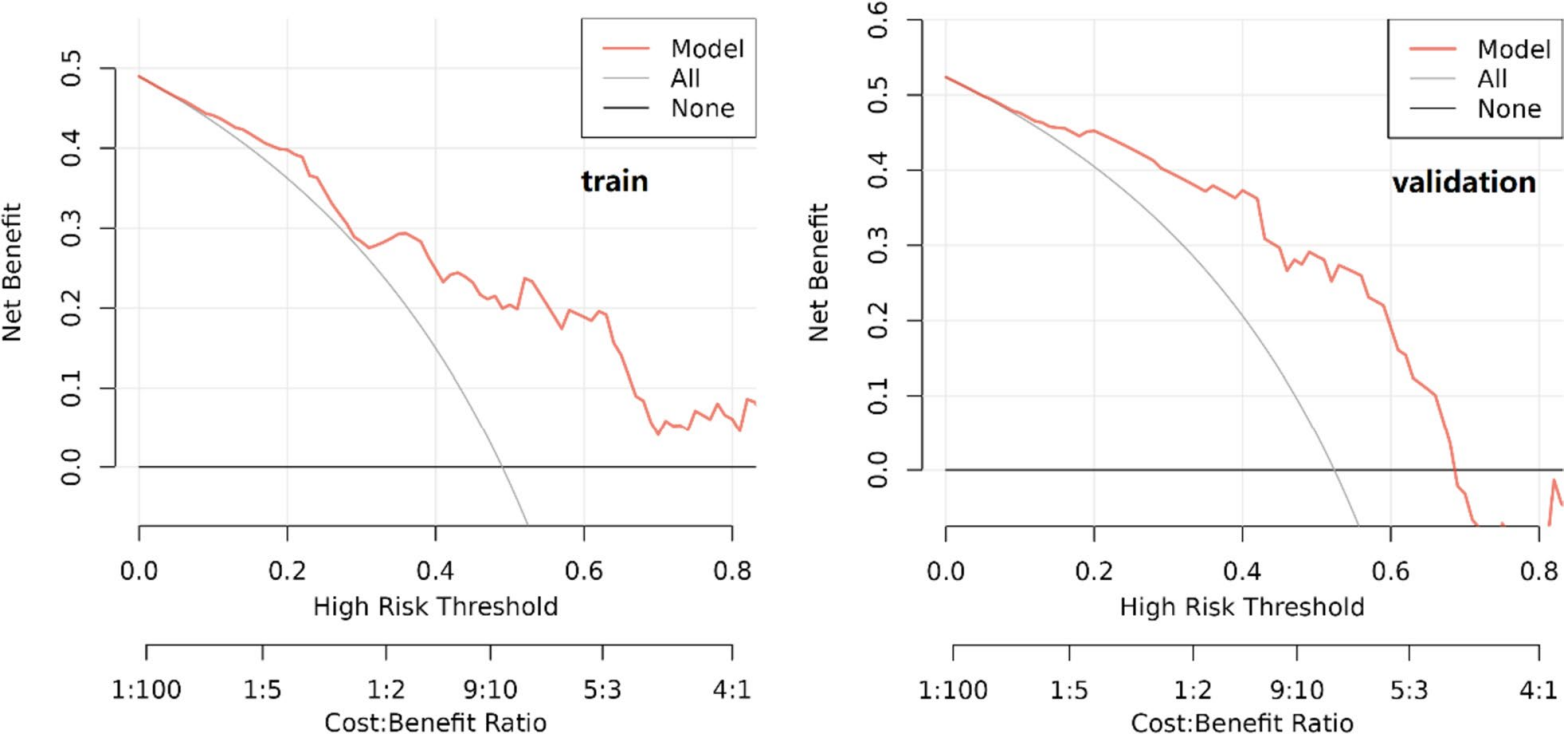
Decision curve analysis of the nomogram

The following Fig. 10 displays the DCA curves related to the nomogram. A high-risk threshold probability indicates the chance of significant discrepancies in the model’s prediction when clinicians encounter major flaws while utilizing the nomogram for diagnostic and decision-making purposes. This research shows that the nomogram offers substantial net benefits for clinical application through its DCA curve.

### Relationship between physical exercise and EEG-specific indicators of depression

Table 5 demonstrates significant correlations between all five EEG metrics selected and SDS scores. However, only δ (C3 + C4) and α1 (F4-F3) exhibited significant correlations with IPAQ scores.

**Table 5:** Correlation between EEG indicators with SDS score and IPAQ score

EEG indicators	SDS	IPAQ
δ (C3 + C4)	*r* = 0.373, *P* < 0.001	*r* = -0.175, *P* = 0.039
δ (F4-F3)	*r* = -0.329, *P* < 0.001	*r* = 0.025, *P* = 0.772
α1 (F4-F3)	*r* = -0.371, *P* < 0.001	*r* = 0.249, *P* = 0.003
α1 (P4-P3)	*r* = -0.185, *P* = 0.029	*r* = 0.130, *P* = 0.125
α2 (F4-F3)	*r* = -0.401, *P* < 0.001	*r* = 0.089, *P* = 0.295

**Fig. 11:**
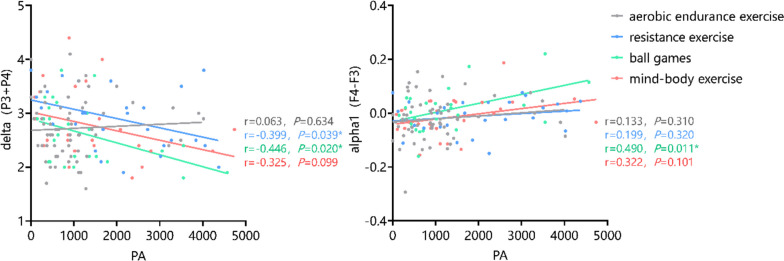
Relationship between physical exercise and EEG: the moderating effect of exercise type

On this basis, a further analysis was carried out to determine whether different forms of physical activity could moderate the correlation between IPAQ scores and two indicators of δ (C3 + C4) and α1 (F4-F3). The results, as shown in Fig. 11, demonstrated that students at the university who were most often engaged in ball games have lower levels of δ (C3 + C4) and higher levels of α1 (F4-F3) with increasing physical activity, whereas those who were most often engaged in resistance sports have lower levels of δ (C3 + C4) with increasing physical activity.

## Discussion

The current study demonstrates that resting EEG exhibits specificity for college students with depressive symptoms. The power values of δ and α2 in the central (C3, C4) and parietal (P3, P4) regions of depressed college students were significantly higher than those of normal college students. And the degree of lateralization of δ, θ, α1, and α2 in the prefrontal regions (F3, F4) of depressed college students was significantly higher than that of normal college students. Most of the δ-wave activity occurs in the temporal and occipital lobes during sleep. In normal adults experiencing extreme fatigue and lethargy, there is a synchronized presentation throughout the cortex [29, 30]. Increased δ-waves can also be associated with impaired brain function or loss of consciousness. It has been found that depressed college students exhibit increased delta-wave activity, slower brain functioning in the waking state, and disrupted regulation of their EEG rhythms, which can disturb sleep cycles [31]. α2-waves are generally related to increased cognitive activity and alertness. Individuals with symptoms of depression may experience imbalances in their neurotransmitters, increased activity within the neurons, or a lack of inhibitory control over excitatory regions of the brain. These factors may result in heightened α2 power values. Increased α-band power values may trigger hypervigilant behavioral performance in individuals with depression [13, 32]. The prefrontal slow wave and fast wave showed lateralization in this population, with the left and right dorsolateral prefrontal cortex corresponding to the F3 and F4 electropole points, respectively. Researchers have discovered that left frontal activity correlates with positive emotions, while right frontal activity correlates with negative emotions, leading individuals to exhibit “approach” and “withdrawal” behaviors, respectively [33]. The lateralization of α-waves might indicate excessive processing of negative emotions and a deficit in emotional regulation [34].

The study indicates that the EEG-based depression recognition model for college students can assess the risk of developing depressive symptoms in subjects with strong discriminatory power. The model is reliable and valid. Among these, the main predictors are δ (C3 + C4), δ (F4-F3), α1 (F4-F3), α1 (P4-P3), and α2 (F4-F3). The depression symptom-specific indices showed consistency, indicating that the above-mentioned indices should be considered as significant observation indices when predicting the occurrence risk of depressive symptoms. The depression identification model in this study confirms the EEG index specificity of college students with depressive symptoms in the measured samples. It also confirms the efficacy of EEG in detecting abnormalities in the brain’s functional activities in depressed individuals. In recent years, scholars have utilized EEG signal data in tandem with data mining classification algorithms to conduct depression recognition research [35–37]. However, the recognition efficacy calls for improvement. Iterative optimization of the model will necessitate a sustained and collective research effort under large sample sizes over a significantly long time with good application prospect.

In this study, it was discovered that increased physical activity was linked to decreased δ power values in the central area (C3 + C4), normalized α1 lateralization in the prefrontal area (F4-F3), and reduced symptoms of depression. Li Chui-kun et al. discovered a reduction in activity of the δ waves in the pontine gyrus located in the occipital lobe, as well as the subparietal lobule situated in the parietal lobe of the left hemisphere of the brain after exercise [38]. Exercise results in energy expenditure, increases cerebral blood flow, and activates the endogenous peptide and amino acid transport systems resulting in the promotion of sleep [39]. As a result, individuals who regularly exercise experience enhanced sleep quality and decreased depressive symptoms [40]. Exercise improves emotional regulation, induces changes in the functional dynamics of the frontal limbic neural network, and results in the right lateralization of the frontal alpha wave [41]. It also serves as a behavior that evokes emotions, mediates the difference in power values between the left and right hemispheres of the brain, and aids the shift from negative to positive emotions in depressed groups of college students [42]. It is worth noting that different types of physical exercise influence the correlation between physical activity level and the indicators of central area (C3 + C4)δ and prefrontal area (F4-F3)α1. The relationship of physical activity level with central area (C3 + C4)δ and prefrontal area (F4-F3)α1 is more robust among university students who play ball games regularly. Additionally, The relationship of physical activity level with central area (C3 + C4)δ is more robust among those who engage in resistance sports regularly. Exercise stimulates the release of neurotransmitters, such as dopamine and endorphins, which enhance positive emotions and provide feelings of pleasure and relaxation. Resistance exercise promotes mRNA expressions, including IGF-1, mTOR, Akt, Syn, and Syp, and improves protein expressions, including IGF-1, IGF-1R, and p-Akt [43]. While participating in ball games, involving teamwork and social interaction, can foster social support system and communication. Such an environment of social support and teamwork can reduce feelings of isolation, enhance social networks, and provide emotional support. It is recommended that individuals who regularly engage in ball games and resistance sports maintain their exercise habits to reap the long-lasting effects of exercise.

Research limitations and recommendations: Several studies have examined the specificity of EEG metrics in individuals with depression. However, the findings have been inconsistent. It is anticipated that future research will collect larger samples of measurements to minimize the impact of individual differences on conclusions. Also, this study employed a subjective questionnaire to assess symptoms of depression. It is important to note that this method does not qualify as a clinical diagnosis and may therefore be prone to subjective biases. The sample population included college students who reported experiencing symptoms of depression. Therefore, the generalizability of these findings may be limited. Additionally, the depression recognition model for college students based on EEG indexes displays greater homogeneity in the distribution of dichotomous dependent variables, necessitating a large-scale model for future confirmation. Finally, this study relied on a subjective questionnaire to gauge physical activity levels, which may have introduced certain subjective biases, and objective measurement tools such as accelerometers are recommended for future use.

## Data Availability

The datasets used and/or analyzed during the current study are available from the corresponding author on reasonable request.
